# Relationship between Mental Health and House Sharing: Evidence from Seoul

**DOI:** 10.3390/ijerph18052495

**Published:** 2021-03-03

**Authors:** Jihun Oh, Jeongseob Kim

**Affiliations:** Department of Urban and Environmental Engineering, Ulsan National Institute of Science and Technology, Ulsan 44919, Korea; wlgnsdl414@unist.ac.kr

**Keywords:** shared housing, young adults, single-person households, residential dissonance framework, GHQ-12

## Abstract

While the association between general housing and mental health has been well documented, little is known about the mental health outcomes of house sharing. As shared housing has been viewed as an economically and socially viable housing option for young adults, a broader understanding of how shared housing affects the residents’ quality of life, including mental health, is needed. In this context, this study aims to provide empirical evidence about the relationship between mental health and house sharing after controlling for residents’ self-selection. We conducted a survey of 834 young single adults living in shared housing and non-shared housing in Seoul, Korea. Then, to control for residential self-selection, we applied the residential dissonance framework. The main findings of this study were two-fold: first, house-sharers with a positive attitude toward shared housing were more likely to respond that their mental health status improved after they started residing in shared housing; second, if young adults are forced to live in shared housing, this could increase the potential risk of social dysfunction of house-sharers. Based on these findings, we suggest policy measures for shared housing, including pre-occupancy interviews, resident behavior codes, and fostering a livable dwelling environment to ensure a healthier life in shared living arrangements.

## 1. Introduction

Along with the increase in the number of single-person households and the proliferation of the sharing economy, shared housing where unrelated adults live together has re-emerged as a viable economic and social housing option for young adults [1]. House sharing has conventionally been seen as an affordable housing option for college students with economic constraints [2]. Recently, house-sharing has been widely adopted by young single-person households, including not only those in the economically disadvantaged group but also those in professional and managerial occupations, to save rent and expand social relationships [3]. Economic constraints are still one of the important considerations for deciding to live in shared housing, but some people choose to live in shared housing in accordance with the needs and demands of their current lifestyle rather than due to economic constraints [1,3]. Further, by allowing more social relationships among housemates and neighbors, shared housing is considered as a community development tool to ensure social capital [4,5].

Home-sharing provides social support from other sharers and caretaking for each other [6]. Oh and Choi [7] found that many of those who choose to live in shared housing anticipate social relationships with their housemates rather than just saving money. Cho et al. [4] reported that shared housing residents express an elevated level of community attachment, which can be a potential resource for community building. However, social opportunities among house-sharers in shared housing do not guarantee positive outcomes in their shared living due to conflicts with housemates and privacy issues [8]. For instance, Rugg [9] points out that some young people with concerns over their safety often end up sharing a house in multiple occupation (HMO) with strangers. Residents in shared housing often speak about the lack of privacy and safety [10]. Ortega-Alcázar and Wilkinson [11] further revealed that for those who were forced to share accommodation due to economic constraints, the shared housing was not perceived as a home but as a place of insecurity and fear. Despite the potential for negative living experiences in shared housing, which can affect the mental health of house-sharers, little is known about the connection between shared housing and residents’ mental health [12].

The relationship between housing and mental health is well documented in the existing literature [13,14]. Swope and Hernandez [14] have provided a holistic conceptual model for health-promoting housing relationships, mainly based on physical conditions, affordability, residential stability, and neighborhood opportunity. It has been widely investigated and confirmed that physical housing quality predicts the resident’s mental health [15,16,17]. Evans et al. [13] focused on psychosocial processes that are thought to link housing and mental health based on five dimensions: identity, control, insecurity, social support, and parenting. To sum up those links, perceptions about where they live [18], an inability to control circumstances [19], residential instability [20], social resources acquired from interactions with housemates [21], and parental control mitigate the process between the physical environment in the house and psychological states.

The mental health of house-sharers can be understood based on the three key mediating factors identified by Evans et al. [13] and Barratt et al. [22]: (1) identity, (2) control, and (3) insecurity. First, identity refers to the symbolic aspects of housing and the local environment, such that living in shared housing impacts a tenant’s identity based on where they live [23]. Second, control refers to the role of housing in providing protection from external conditions. Green and McCarthy [8], for example, found that social interaction with other tenants was shown to be associated with stress, anxiety, and insecurity, especially when it was unwanted. Other uncontrollable situations such as noise or housing maintenance also contribute to the residents’ mental distress [22]. Third, insecurity has been associated with residential insecurity such as having to move frequently or relational insecurity such as anxiety about safety, hygiene, and crime. In this regard, Stewart et al. [21] suggested that mental distress from home-sharing mainly originates from the insecurity of short-term tenancy and community transiency. Among the 2657 rooms available for shared housing in Seoul in 2018, 24.6% of them had a lease term of less than six months [24].

Despite the accumulated knowledge on the impact of the house environment on residents’ mental health and the efforts to explore how shared housing is linked to mental health, empirical evidence for their causal relationship is surprisingly scarce. Ahrentzen [25] examined the health consequences of shared housing in terms of physical, psychological, social, and economic aspects. Page [19] emphasized how living in houses in multiple occupations (HMOs) often negatively impact mental health, and that adults living in temporary accommodation suffer from increased levels of depression, domestic violence, alcoholism, family stress, and relationship breakdown. Green and McCarthy [8] found that the poor quality of shared housing and the behavior of other tenants are associated with stress, anxiety, and insecurity. These studies provide several implications for mental health outcomes resulting from co-living with strangers. However, empirically, little is known whether living in a shared housing results in significant mental health outcomes compared to living in other conventional (independent) housing.

Understanding the needs of single young adults in the context of shared housing is essential for solving the housing challenges currently facing this demographic group [1]. Similarly, understanding the consequences of shared housing on the sharers’ health should precede the development of appropriate housing strategies to manage them. Therefore, this study aimed to explore the effect of shared living on the sharers’ mental health. Specifically, the study addresses two research questions: (1) Does house-sharing improve the mental health status of house-sharers? and (2) If so, after controlling for other factors, do house-sharers show significantly better mental health status than non-sharers?

To answer these research questions, we conducted a survey of 834 single young adults in Seoul, Korea, 334 living in shared housing and 500 living in non-shared housing) and analyzed their perception of shared housing, current residence characteristics, and mental health status. Based on quantitative analysis, this study provides empirical evidence for the effect of house-sharing on mental health. The next section explains how the analytical variables to measure mental health were constructed and the analytical strategy used to address the self-selection issue. This is followed by the results section, then, the main findings are discussed and some policy and managerial suggestions for shared housing are given.

## 2. Materials and Methods

### 2.1. Data and Variables

All the data and variables used in the analysis were obtained from the survey summarized in Table 1. We targeted young single-person in renter households in Seoul, the capital city of Korea. We chose Seoul as the study area because of its relatively expensive housing costs compared to other regions, which has resulted in the city suffering from affordability issues, especially for young adults. According to an investigation by the City of Seoul [26], 21.3% of those aged below 40 had experienced housing cost burden. A growing number of young single persons are delaying marriage, which is further increasing housing demand, thus aggravating the housing affordability issue. As one of the solutions to the housing demand and affordability issues among young adults, many for-profit and non-profit organizations have started to provide shared housing for young adult groups [1,10]. In this context, Seoul is a suitable case city to study shared housing. The survey was conducted by Hankook Research, one of the top professional social survey companies in Korea, between 24 August and 5 October 2018.

The survey questionnaires included socio-demographic attributes, current residence attributes, and other indicators to represent the quality of life including mental health-related measures among young single-person households. A description of each of these variables is presented in Table 2. For residential satisfaction, participants were asked to respond to the statement, “Overall, I am satisfied with my current residence.” Residential satisfaction was identified as one of the important mediating factors between dwelling condition and mental health, mainly in terms of psychological well-being [27,28]. For the indicators for mental health, we considered two aspects of mental health: psychological well-being and mental distress. Barratt et al. [22] argued that mental health as a residence outcome cannot be fully understood using a single indicator and Bond et al. [29] suggested that concepts such as mental well-being can also be meaningful if we are to understand how housing affects mental health in the general population. Therefore, we measured both mental health status improvement and distress aspects of mental health. We measured mental health status improvement using one questionnaire item, “Overall, my mental health status has improved since I started residing at the current residence.” If the residents selected “3” or higher on a four-point Likert scale, we classified them as having improved mental health status. To measure the level of mental distress, we utilized the General Health Questionnaire (GHQ)-12 items (the Korean version), which has been verified for accuracy and brevity by Cano et al. [30]. In their study on the validity of the Korean version GHQ-12, Park et al. [31] also revealed that the Korean translated version shows high internal consistency in identifying psychological distress and it has a two-factor structure with an anxiety disorder and social dysfunction. More specifically, to detect a potential disorder in mental health status, we first calculated the average mental distress level in the related GHQ-12 items for anxiety and depression and social dysfunction. Then, for better discrimination of multiple psychiatric disorder as suggested by Kim et al. [32], we used a very conservative cut-off at 2/3 rather than 1/2 to identify the existence of disorder in each mental distress factor. Last, to control the baseline effect of personality on mental health status, we utilized four relevant questionnaires from the Myers–Briggs Type Indicator (MBTI) test. These included, for example, agreement with statements such as, “I tend to have a wide range of friendships with people” or “I tend to get along easily with other people,” with the answers “yes” or “no”. Cronbach’s α for these items was 0.83, which indicates the high internal consistency of the used questionnaires. Because of the length of the survey questionnaire, we could not use all of the questionnaire in the MBTI test. Thus, it is important to note that the validity of the construct used to measure personality should be systematically verified in future studies while the current one has high internal consistency. Personality has been widely investigated as a potential mediator between social support and mental health [33]. Therefore, this study hypothesized that an extroverted personality may be connected with high engagement in social interaction and this further reduces the probability of potential mental disorder (especially in social dysfunction).

### 2.2. Methods of Analysis

The procedure for data analysis was as follows. First, the overall characteristics of shared housing residents were presented using descriptive statistics. Next, we compared the indicators for the mental health status of shared housing and non-shared housing residents using graphs. Further, by applying the residential dissonance framework explained in the following paragraphs, logistic regression models were constructed to examine whether the differences in mental health status originate from current shared living experiences when residents’ self-selection was addressed. Last, in the discussion section, we suggest some explanations and links for the impact of shared living on mental health, mainly focusing on the psychosocial processes suggested by Barratt et al. [22].

To provide more robust empirical evidence of a causal relationship between shared living and mental health outcomes, residents’ self-selection issues should be addressed methodologically [34]. It is difficult to separate the influence of housing on mental health as distinct from other socio-demographic and preferences of the respondents [35]. For example, people living in shared housing may have a healthy mental health status because the living experiences in shared housing either enhance psychological well-being or reduce mental distress, but at the same time, people who have a healthy mental status may tend to choose to live in shared housing. To address the residential self-selection of house-sharers, this study utilized the “residential dissonance framework” proposed by Schwanen and Mokhtarian [36]. Although the residential dissonance framework has mainly been utilized to examine the effects of self-selection on residential location choice and travel behavior [37], it can also be a useful methodological approach for shared housing studies as shown in Cho et al. [4].

The residential dissonance framework assumes that there could be a considerable mismatch between preferred residence type and actual residential choice (i.e., current residence) for several reasons, such as budget constraints, which force one to live in a certain residence [38]. Based on that assumption, it can be reasonably expected that living outcomes in residents who prefer to live in shared housing and residents who are forced to live in shared housing might be different [34]. Therefore, this study hypothesized that the living outcome in terms of mental health differs between consonant and dissonant residents living in both shared housing and other non-shared housing. Following the configuration suggested by Cho et al. [4], four types of residents were defined as follows:

1. Consonant shared housing residents (CS): those who reside in shared housing and have positive attitudes toward sharing residential space.
2. Dissonant shared housing residents (DS): those who reside in shared housing and have negative attitudes toward sharing residential space.
3. Dissonant other housing type residents (DO): those who reside in other (non-shared) residential types and have positive attitudes toward sharing residential space.
4. Consonant other housing type resident (CO): those who reside in other (non-shared) residential types and have negative attitudes toward sharing residential space.

This study measured residential preference as to whether the residents had a positive or negative attitude toward shared living. In more detail, the attitude toward shared living was calculated as the average of responses to eight survey items on a four-point Likert scale that asked for the perception on shared housing. The items were as follows: “it helps to save housing cost,” “it helps to build a new social relationship,” “it helps to reduce loneliness,” “it provides better location and facilities,” “it provides better safety and security,” “it does not guarantee privacy,” “it has concerns about social conflicts with housemates,” and “it has noise and cleanliness issues.” Considering the reverse scoring in the last three questions, if the average value was higher than the median value of the four-point Likert scale (2.5), we assumed that the resident had a positive attitude toward sharing residential space. In this way, by differentiating between those who had a preference to live in a certain housing type and others who did not, the living outcome from shared living can also be categorized as the impact of a shared housing environment or the impact of residential preference. For example, if both consonant and dissonant shared housing residents showed better mental health status than both consonant and dissonant non-shared housing residents, it indicated that the living experiences under the current shared housing environment substantially enhanced the residents’ mental health regardless of whether they chose or were forced to live there. On the contrary, there could be a case where only consonant shared housing residents and dissonant non-shared housing residents show enhanced mental health outcomes compared to other resident types. In this case, it is hard to say that the shared living experiences enhanced the residents’ mental health status. Rather, it indicates that mentally healthy people might prefer shared housing. The opposite situation is also possible. As suggested by Green and McCarthy [8], shared living experiences can negatively affect mental health status due to conflicts among housemates or lack of privacy.

## 3. Results

### 3.1. Characteristics of Shared Housing Residents

Table 3 shows the overall characteristics of shared and non-shared housing residents using a two-tailed t-test to compare the mean value between them. Shared housing residents tended to have more positive attitudes toward shared living than general housing residents. There were no significant differences in the attitudes toward shared living based on socio-demographic attributes such as gender and income level, except for the tendency that younger residents had a more positive attitude toward shared living. The youngest age group (20–24 years) was more likely to live in shared housing. This is consistent with the characteristics of sharers identified in previous studies [1,4]. With regard to gender, shared housing had a relatively higher number of male residents than other housing types, which is also consistent with the findings of Woo et al. [10] that young single males were significantly more likely to choose shared housing than young single females. It has been widely shown that single females tend to be more sensitive to safety and crime issues [39], which might make them reluctant to share living spaces with strangers.

About 90% of shared housing residents and 62% of non-shared housing residents responded that they were paying rent monthly. In the same context, the average residence period of house-sharers was about one and a half years, which was relatively shorter than two years and four months for non-house sharers. In addition, contrary to what is generally believed, the cost of living in shared housing was not significantly lower than other housing types (only about 50,000 KRW).

The residents in shared housing were more likely to report that their mental health status had improved since they started residing in their current residence, but they had nearly similar ratios to those who showed signs of potential disorder in mental health status. Extroverted people also tended to choose shared housing more than introverted people. However, it should be noted that there exists a dissonance in current residence so that the perception, behavior, and resulting living outcome of current residents might differ based on whether the resident was consonant or dissonant. Thus, in the next section, using the residential dissonance framework, we will discuss the results on whether shared housing residents showed substantially better or worse mental health status than others, and whether the difference in mental health remained statistically significant after controlling for other factors using a logistic regression model.

### 3.2. Mental Health Outcomes of House-Sharing

**Figure 1:**
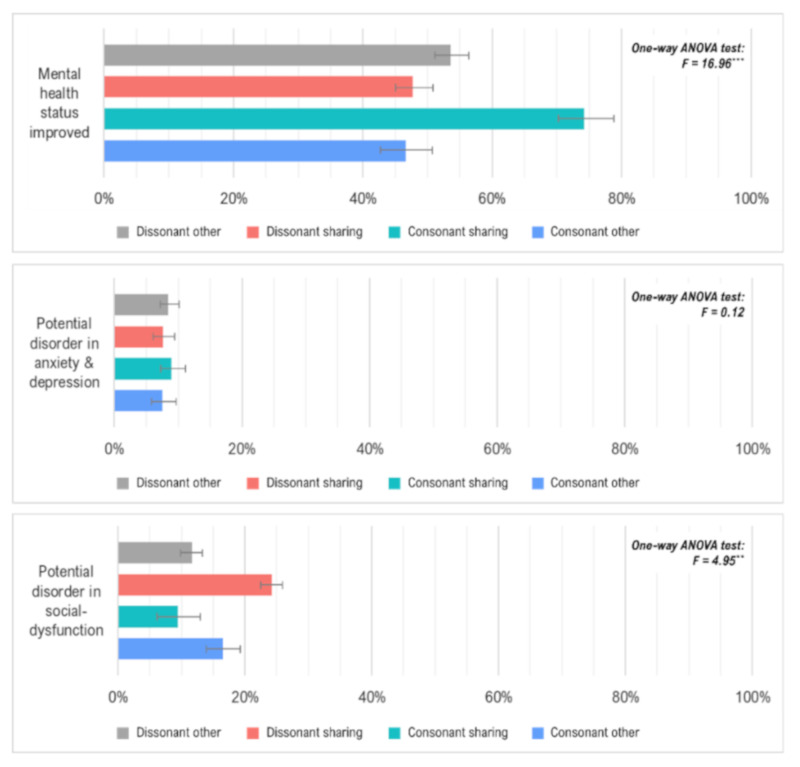
Mental health status by residential dissonance type. Note: *, **, *** refer to the statistical significance at the 5%, 1%, and 0.1% level. The error bars in the graph refer to the standard errors.

The mental health status for each indicator by residential dissonance type using the one-way analysis of variance (ANOVA) test assuming non-equal variance are presented in Figure 1. To ensure the non-equal variance, we conducted a Levene’s test using R software to check that the variances were non-equal for each residential dissonance type before applying the ANOVA tests. The results for all of the mental health indicators, showed that the null hypothesis of equal variance had been rejected with a significance level of 95%. For the ratio of those who reported that their mental health status had improved since they started residing at the current residence, the consonant shared housing residents (CS) showed the highest level compared to the others. The dissonant shared housing residents (DS) and consonant other housing residents (CO) who had negative attitudes toward shared living showed a relatively lower ratio of mental health status improvement than the other two residential dissonance types. This suggests that mental health status improvement might differ based on personal preference regarding shared housing, rather than the living experiences under shared housing. Put differently, even though the shared housing residents generally showed better indication of mental health status improvement, this might be limited to those who chose (not forced) to live in shared housing. 

In terms of mental distress, there were significant differences in potential disorder in social dysfunction based on residential dissonance type, while this was not the case for potential disorder in anxiety and depression. More specifically, the differences in potential disorder in social dysfunction showed a similar value to that of mental health status improvement, except for its direction. In other words, the preference for shared housing was negatively correlated with the ratio of potential disorder in social dysfunction, as it was positively correlated with the ratio of mental health status improvement.

**Figure 2:**
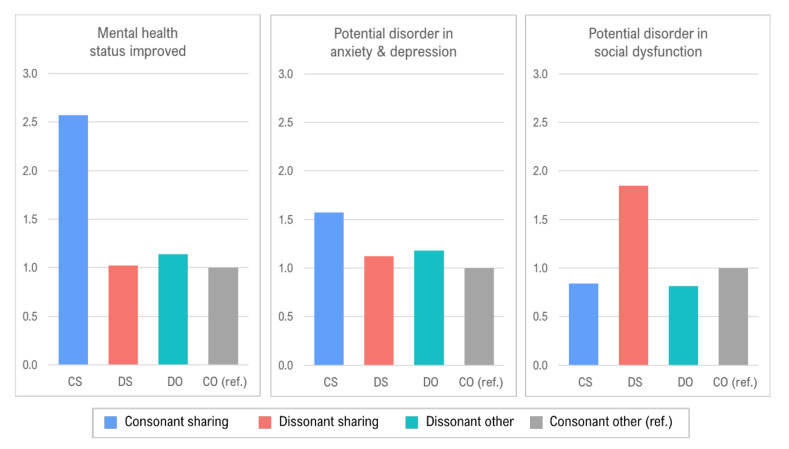
Odds ratios for each mental health status by residential dissonance type.

To further examine whether the above differences would remain statistically significant when holding other variables constant, we constructed three logistic regression models where the dependent variables were the mental health indicators shown in Table 4, and the graphical results of the regression results focusing on indicators of mental health status are presented in Figure 2. Only consonant shared housing residents (CS) were more likely to show mental health status improvement. This result is consistent with Green and McCarthy [8] in showing that the house-sharers who wanted the shared-living arrangements could enjoy the social relationships and be less stressed in the social interactions in shared housing. The residents who had higher residential satisfaction and an extroverted personality also reported that their mental health status had improved in their current residence. For the mental distress indicators, there were no significant differences in terms of anxiety and depression except for residential satisfaction. As suggested by Barratt et al. [22], residential satisfaction may be related to psychosocial mitigation factors, such as controlling residential environments and the security of houses, in determining the mental health outcomes of residents. Further discussion about this issue is followed in the next section by comparing the perception between consonant and dissonant house-sharers.

On the other hand, several factors were found to have a significant impact on a potential disorder in social dysfunction. Specifically, those in the youngest age group (20–24), satisfied with their current housing environment, or who had an extroverted personality were less likely to show signs of mental disorder in social dysfunction. Most importantly, the dissonant shared housing residents (DS) had a significantly higher probability of being socially dysfunctional. These results are consistent with the findings of Green and McCarthy [8], Rugg [9], and Ortega-Alcázar and Wilkinson [11] that mental health outcomes such as mental improvement or potential disorder in social dysfunction were substantially correlated with a residential preference for shared housing. In short, the mental health of house-sharers with a positive attitude toward house-sharing could be improved; however, there is a potential risk of social dysfunction if dissonant house-sharers reside in shared housing.

## 4. Discussion

The main findings of this study are two-fold: first, the consonant shared housing residents generally showed a better mental health status than the other non-shared housing residents with regard to mental health improvement; and second, there is a possibility that shared housing residents can suffer from the disorder in social dysfunction if they do not have a preference for shared living. In terms of the association between shared living and mental health outcomes, Barratt et al. [22] proposed three psychosocial mitigating processes that link them: identity, control, and insecurity. Accordingly, it can be reasonably speculated that as there were considerable differences in mental health outcomes between consonant and dissonant shared housing residents, there would also be significant differences in those psychosocial mitigating factors based on residential dissonance. Although the statistical test for their causal link is not the main focus of this study, the differences will guide us in providing a more plausible interpretation of the results.

To compare psychosocial mitigating processes between consonant and dissonant shared housing residents, we measured three constructs for each process in reference to Barratt et al.’s [22] configuration. First, identity refers to how the residents perceive their current residence and community, which is related to concepts such as a sense of belonging or a sense of community [40]. Further, identity in the context of shared housing is related to social support in that the formation of positive and supportive social relationships with housemates can boost self-esteem, which results in an enhanced sense of identity [41]. The construct for social support was measured using the average of four survey items on a four-point Likert scale (higher points mean stronger social support): (1) “I feel comfortable when talking with housemates,” (2) “I can ask housemates for help without any burden,” (3) “I know my housemates well and they know me well,” and (4) “I often talk to housemates when I make an important decision.” Next, whether residents have control over their living condition can also affect their mental health status. Here, we measured control over housing conditions using a survey item asking about the noise and state of cleanness in their current residence on a four-point Likert scale (higher points mean a worse condition). Lastly, for insecurity, we measured safety as the security and safety condition of the current residence on a four-point Likert scale (higher the better condition).

Table 5 shows a comparison of the results for each construct between consonant and dissonant shared housing residents using a two-paired t-test. In all the variables, the differences by residential dissonance type were highly significant. That is, stronger social support by housemates and better dwelling conditions in terms of both housing maintenance and security were more likely to be found among consonant shared housing residents. This suggests that shared living can lead to improvement in mental health status, especially for those who have a strong sense of identity and feel secure in their home, but at the same time, it can lead to the occurrence of a disorder in social dysfunction when there is no proper control over the dwelling condition. However, these simple statistical tests do not confirm any causal relationships between the psychosocial mitigating process and mental health outcomes of house-sharers. The mental health outcomes by residential dissonance types could originate from not only their residential attitudes but also their experiences in the residences. Therefore, more systematic research to examine the role of psychosocial mitigating processes in mental health outcomes should be conducted with an emphasis on the housing quality of shared housing and tenants’ behaviors.

## 5. Conclusions

Shared housing has maintained its popularity as a policy option for affordable housing supply. In the UK, the HomeShare UK scheme was launched in 1993 to improve well-being (specifically mental health) by reducing loneliness and isolation and there were 27 HomeShare providers in 2017 [42]. The Korean government also makes an effort to provide shared housing as an economically and socially viable solution to the affordability issue faced by young single-person households. Recently, for example, the Seoul municipal government decided to provide various subsidies for shared housing startups to facilitate the supply of shared housing [1]. To set up adequate measures to achieve these policy goals, the effects of living in shared housing on the residents need to be understood first. Nevertheless, the consequences of house sharing on mental health have been little studied. This study aimed to contribute to filling the gap by examining the relationship between house sharing and mental health status by considering the tenant’s residential dissonance type, and found that house sharers show better mental health indicators in terms of mental health improvement and less probability of being in danger of social dysfunction, especially for those who voluntarily chose to live in shared housing.

The findings from this study have several practical implications for the allocation and management of shared housing as follows. First, the study shows that uncontrollable environments such as unwanted social interaction with strangers or poor housing maintenance conditions, and residential insecurity may result in an unhealthy mental status. Thus, a pre-occupancy interview before moving into a shared house could be helpful to further promote improvement in mental health status and to mitigate the risk of disorder in social dysfunction of house-sharers. The interview might inform or filter dissonant shared housing residents who might not want to live there. Providing an independent affordable housing option for those who do not prefer unintended social interaction or community activities to prevent mental distress should be considered, too. Second, it shows that the behavior of other tenants seems to be related to mental health status. As Nasreen and Ruming [43] found, the social elements (e.g., conflicts with housemates) within shared living affect the home-making behavior of the sharers, thus, a properly and systematically designed scheme for managing tenant’s behavior would be needed. For example, a comprehensive behavior code regarding aspects such as the division of work, community activity, and regulation on anti-social behavior needs to be organized to harmonize social interactions among the sharers and provide a controllable and secure environment. Third, residential satisfaction, that is satisfaction on the quality of shared housing, was significantly and positively correlated with mental health indicators. This indicates that fundamentally, fostering a livable dwelling environment should be prioritized. As the impact of physical housing conditions on mental health has already been evidenced in the body of literature [14], attention to policy and efforts to sustain the quality of shared housing should be continued.

This study provides empirical evidence regarding the relationship between house-sharing and mental health. To function as a sustainable housing option presently and in the future, a broader understanding of the living outcomes of shared housing, including mental health, are necessary to build more successful and appropriate policy measures. Similar to this study, which extends the knowledge of how shared housing affects the mental health of its residents, the socio-economic and health outcomes of shared housing should be explored in different cultural and country contexts to provide more generalizable findings. To provide more robust findings regarding health outcomes and residential environments, pre-and post-test research design, and an understanding of the role of psychosocial mitigation factors should be employed. This study presents several theoretical relationships between living in shared housing and mental health outcome. As Kenyon and Heath [3] noted, for young adults, shared housing is no longer viewed as only an affordable housing option but also an opportunity to expand one’s social relationships. Therefore, continued studies and policy efforts to ensure positive social interactions in shared housing could lead to a better quality of life for house-sharers.

## Figures and Tables

**Table 1:** Survey design.

Type	Residents in Shared Housing	Residents in General Housing (Not Sharing)
Survey target	Single persons aged between 20 and 39 living in shared rental housing in Seoul	Single persons aged between 20 and 39 living in general (non-shared) rental housing in Seoul
Sampling	Stratified sampling based on the number of shared housing units in five living zones in Seoul	Stratified sampling based on the number of single-person renter households in five living zones in Seoul Sub-age groups were considered in the stratified sampling
Survey Method	Face-to-face survey	Online survey
Sampling Size	334	500

**Table 2:** Description of variables.

Variables		Description
Socio- Demographic Attributes	Gender	(1) Male, (2) Female
	Age	(1) 20–24, (2) 25–29, (3) 30–34, (4) 35–39
	Income	Average monthly income including support from parents (unit: 1000 KRW)
Residence Attributes	Tenure type	(1) Monthly rent (2) “*Jeonse*” (a large amount of deposit instead of paying monthly rent)
	Housing cost	Average monthly housing cost burden including rent and maintenance fee (unit: 1000 KRW)
	Residential satisfaction	Overall satisfaction with current residence (on a four-point Likert scale)
	Residence period	Total residence period in current residence (unit: month)
Mental Health Indicators	Mental health status improved	Response to “Mental health status has improved since I lived in the current house” (i.e., agreement of that statement was 3 or more on a four-point Likert scale)
	Potential disorder in anxiety & depression	Show signs of potential disorder in anxiety and depression (i.e., average anxiety and depression level was above 3 on a four-point Likert scale)
	Potential disorder in social dysfunction	Show signs of potential disorder in social dysfunction (i.e., average social dysfunction level was above 3 on a four-point Likert scale)
Personality		(1) Introvert, (2) Extrovert

**Table 3:** Descriptive statistics for shared housing and other housing residents.

Variables		Shared Housing		Other Housing		*t* Statistics
		Mean	Std. dev.	Mean	Std. dev.	
Attitude toward sharing space		2.71	0.37	2.43	0.34	10.74 ***
Socio-demographic attributes	Gender (Male = 0, Female = 1)	0.60	0.49	0.73	0.45	−3.89 ***
	Age group: 20–24	0.30	0.46	0.20	0.40	3.45 ***
	Age group: 25–29	0.31	0.46	0.33	0.47	−0.35
	Age group: 30–34	0.23	0.42	0.29	0.45	−1.83
	Age group: 35–39	0.15	0.36	0.19	0.39	−1.46
	Monthly income (1000 KRW)	2198.98	1258.89	2289.18	1212.96	−1.03
Residence attributes	Tenure type (Monthly rent = 0, Yearly rent = 1)	0.10	0.31	0.38	0.49	−10.09 ***
	Housing cost (1000 KRW)	549.00	278.31	599.86	286.27	−2.16
	Residential satisfaction (four-point Likert scale)	2.94	0.62	2.79	0.62	3.40 ***
	Residence period (month)	18.51	19.89	27.59	25.69	−5.74 ***
Mental health indicators	Mental health status improved (Yes = 1, No = 0)	0.64	0.48	0.49	0.50	4.33 ***
	Potential disorder in anxiety and depression (Yes = 1, No = 0)	0.08	0.28	0.08	0.27	0.30
	Potential disorder in social dysfunction (Yes = 1, No = 0)	0.15	0.36	0.15	0.36	0.11
Personality (Introvert = 0, Extrovert = 1)		2.31	1.48	1.80	1.53	4.79 ***
N		334		500		

Note: *, **, *** refer to the statistical significance at the 5%, 1%, and 0.1% level, respectively.

**Table 4:** Logistic regression models for estimating mental health outcomes (*n* = 834).

Variables		Mental Health Status Improved		Potential Disorder in Anxiety and Depression		Potential Disorder in Social Dysfunction	
		Odds Ratio	z Value	Odds Ratio	z Value	Odds Ratio	z Value
Residential Dissonance	Consonant shared housing residents (CS)	2.570	4.382 ***	1.573	1.271	0.843	−0.553
	Dissonant shared housing residents (DS)	1.022	0.101	1.124	0.291	1.848	2.285 *
	Dissonant other housing residents (DO)	1.142	0.65	1.178	0.454	0.814	−0.689
	Consonant other housing residents (CO, ref.)						
Socio- Demographic Attributes	Gender (Male = 0, Female = 1)	0.882	−0.753	1.018	0.064	1.252	1.001
	Age group: 20–24	0.720	−1.171	1.043	0.092	0.373	−2.514 *
	Age group: 25–29	0.829	−0.787	0.962	−0.1	0.749	−0.94
	Age group: 30–34	1.003	0.011	0.635	−1.135	0.812	−0.705
	Age group: 35–39 (ref.)						
	Monthly income (1,000 KRW)	1.008	1.057	0.996	−0.325	0.980	−1.785
Residence Attributes	Tenure type (Monthly rent = 0, Yearly rent = 1)	1.062	0.334	1.313	0.881	0.688	−1.5
	Housing cost (1000 KRW)	1.001	0.333	1.001	1.461	1.001	−0.103
	Residential satisfaction (four-point Likert scale)	2.237	6.099 ***	0.830	−1.896	0.682	−2.384 *
	Residence period (month)	0.997	−0.874	1.004	0.788	1.007	1.821
Personality (Introvert = 0, Extrovert = 1)		1.123	2.300 *	0.924	−0.884	0.755	−3.911 ***
(Intercept)		0.080	−5.065 ***	0.102	−2.933 **	1.438	0.583
Pseudo R-squared (Maximum-Likelihood)		0.117		0.009		0.066	

Note: *, **, *** refer to the statistical significance at the 5%, 1%, and 0.1% level, respectively.

**Table 5:** Comparison of psychosocial mitigating factors by residential dissonance type.

Psychosocial Mitigating Processes	Constructs	Consonant Shared Housing Residents	Dissonant Shared Housing Residents	*t* Statistics
Identity	Social support by housemates	2.63	2.09	7.49 ***
Control	Noise and state of cleanness in current residence	2.70	3.06	−4.85 ***
Insecurity	Security and safety condition of current residence	3.14	2.45	10.00 ***

Note: *, **, *** refer to the statistical significance at the 5%, 1%, and 0.1% level, respectively.

## Data Availability

The data presented in this study are available on request from the corresponding author.
